# Fixed Combination for the Treatment of Dyslipidaemia

**DOI:** 10.1007/s11883-023-01142-x

**Published:** 2023-09-16

**Authors:** Nicola Ferri, Massimiliano Ruscica, Raul D. Santos, Alberto Corsini

**Affiliations:** 1 Department of Medicine (DIMED), University of Padova, Via Giustiniani 2, 35128 Padua, Italy; 2 Veneto Institute of Molecular Medicine (VIMM), Via Orus 2, 35129 Padua, Italy; 3 Department of Pharmacological and Biomolecular Sciences “Rodolfo Paoletti”, University of Milan, 20133 Milan, Italy; 4 Department of Cardio-Thoracic-Vascular Diseases – Foundation IRCCS Cà Granda Ospedale Maggiore Policlinico, Milan, Italy; 5 Lipid Clinic, Heart Institute (InCor), University of São Paulo, São Paulo, Brazil; 6 Hospital Israelita Albert Einstein, São Paulo, Brazil

**Keywords:** Statins, Bempedoic acid, Ezetimibe, Fenofibrate, Combinations, Polypill

## Abstract

**Purpose of Review:**

It is clear from epidemiological studies that patients at high and very-high risk of atherosclerotic cardiovascular diseases (ASCVD) risk do not reach lipid guideline–recommended targets. Thus, fixed-dose combinations of statins/ezetimibe, bempedoic acid/ezetimibe and statins/fibrates may represent a further armamentarium in the field of lipid-lowering approaches in these individuals.

**Recent Findings:**

The combination therapy of moderate-intensity statin with ezetimibe is not inferior to high-intensity statin monotherapy in reducing cardiovascular outcomes. Drug discontinuation or dose reduction is inferior with fixed-dose combination. The fixed-dose combination of bempedoic acid with ezetimibe is superior to bempedoic acid in monotherapy in lowering LDL-C and in reducing high-sensitivity C-reactive protein concentrations. The combination fenofibrate with atorvastatin is superior to monotherapies in lowering triglycerides.

**Summary:**

Lipid-lowering fixed-dose combinations may guarantee a higher therapy adherence, representing a better approach to control plasma lipids and thus ameliorate ASCVD burden. Additional studies will define the advantages on cardiovascular outcomes in high and very high-risk patients.

## Introduction

Cardiovascular diseases (CVD) are a leading cause of death worldwide, and several modifiable and unmodifiable risk factors contribute to this burden of disability and mortality [1]. Effective cardiovascular prevention relies on appropriate strategies to control risk factors within the frame of unmodifiable traits. Dyslipidaemia represents the most relevant modifiable factor of atherosclerotic cardiovascular diseases (ASCVD). Current guidelines provide clear indications for the targets that should be reached, and sustained over a life-long period, for the low-density lipoprotein cholesterol (LDL-C) [2]. While combination therapies have been widely used in the management of conditions such as hypertension and type 2 diabetes mellitus, this pharmacological approach has been used to manage dyslipidaemia, only recently.

The rationale of developing fixed drug combination is to improve therapy efficacy by minimizing the incidence of adverse side effects. Fixed-dose combination therapies can substantially complement and ameliorate current strategies for reducing the global burden of ASCVD risk [3]. In addition, there is a consensus that an effective way to improve adherence to treatment is the simplification of the therapy (i.e. reduction of the number of tablets to be taken daily) [4].

From the pharmacological point of view, the drugs used as fixed combination for reducing the LDL-C should act with different mechanism of action, providing an additive hypocholesterolemic effect. These drugs should have similar elimination half-life times so they could be administered with the same posology and should not interact in their pharmacokinetic profile.

Since their approval in 1987, the hydroxy-methyl-glutaryl CoA (HMG-CoA) reductase inhibitors, statins, have represented the first line of treatment for controlling hypercholesterolaemia. A robust number of randomized controlled trials (RCTs) have shown that statins, by lowering circulating LDL-C reduce the absolute risk of CVD and mortality. Each mmol/L (38.7 mg/dL) reduction in LDL-C is associated with a relative risk reduction of approximately 22% [5]. This association is maintained up to very low levels of LDL-C [6, 7]. This evidence is confirmed in genetic studies showing that beyond the paradigm “the lower the better”, the earlier the reduction, the greater the cardiovascular benefit [8]. In fact, a floor profit level has not yet been identified and larger early LDL-C reduction and more intensive statin therapy after myocardial infarction have been associated with a reduced hazard of all CV outcomes and all-cause mortality in real-world setting [9]. However, treatment with this class of drugs may not be sufficient to reach the recommended LDL-C goals for high and very-high CV risk patients [2, 10] and for controlling the triglyceride (TG) levels in the mixed hyperlipidaemias.

Within this context, alarming are the data of the DA VINCI [11••] and SANTORINI [12] studies showing that, among European patients at high and very high-risk for ASCVD, only a few percentage (roughly between 20 and 33%) reach their LDL-C targets. These observational studies have clearly documented the gaps between clinical guidelines and the real-world lipid management which have been exacerbated after publication of the new 2019 LDL-C targets [11–14].

The fixed combination of statins with the Niemann-Pick C1-Like 1 (NPC1L1) inhibitor ezetimibe and with the peroxisome proliferator-activated receptors-α (PPAR-α) agonists, fibrates, are widely used to treat dyslipidaemias. More recently, the inhibitor of the adenosine triphosphate citrate lyase (ACLY), bempedoic acid, has been approved as monotherapy or in combination with ezetimibe to reduce LDL-C. Lipid-lowering combination therapies are also recommended for the management of patients with heterozygous familial hypercholesterolaemia [15].

The choice of the appropriate hypocholesterolemic therapy is of pillar importance considering that the relative risk reduction of major vascular events is independent of the starting levels of LDL-C or the presence of diabetes or chronic kidney disease (CKD) but proportional to the amount of absolute cholesterol lowering. Indeed, the combination of appropriate treatment intensity and adherence associates with greater reduction of LDL-C and cardiovascular outcomes in patients at high risk [14•].

A broader approach for controlling CV risk is represented by the development of a polypill containing combination of antiplatelet, hypocholesterolemic and antihypertensive medications. This strategy improves the adherence and results in a significantly lower risk of major adverse CV events than usual care [16–22].

On this review, we provide the pharmacological rational, the pharmacokinetic and pharmacodynamic profiles, and the clinical efficacy and safety of the most widely used and innovative fixed drug combinations for the treatment of dyslipidaemias.

## Fixed Combination of Statins and Ezetimibe

Genetic, observational, and interventional studies have demonstrated that LDL-C, and other ApoB-containing lipoproteins, have a direct casual role in the development of ASCVD [23]. The relative CVD risk reduction is proportional to the absolute change in LDL-C, irrespective of the drug(s) used to achieve such change [5, 24]. Ezetimibe is the only available drug acting by inhibiting the (NPC1L1) transporter and thus reducing intestinal cholesterol absorption [25]. Ezetimibe is rapidly absorbed and extensively conjugated to its pharmacologically active glucuronide derivative. Mean peak plasma concentrations (C_max_) are observed within 1–2 h for ezetimibe-glucuronide and 4–12 h for ezetimibe [26]. In monotherapy, 2 weeks of treatment with 10 mg of ezetimibe reduced LDL-C and total cholesterol (TC) by 20.4% and 15.1%, respectively [27]. In response to the inhibition of intestinal absorption of cholesterol, ezetimibe activates a compensatory increase (+89%) of hepatic cholesterol synthesis [27], thus providing a rational for using a fixed oral combination with statins, that inhibit cholesterol synthesis in the liver.

**Fig. 1:**
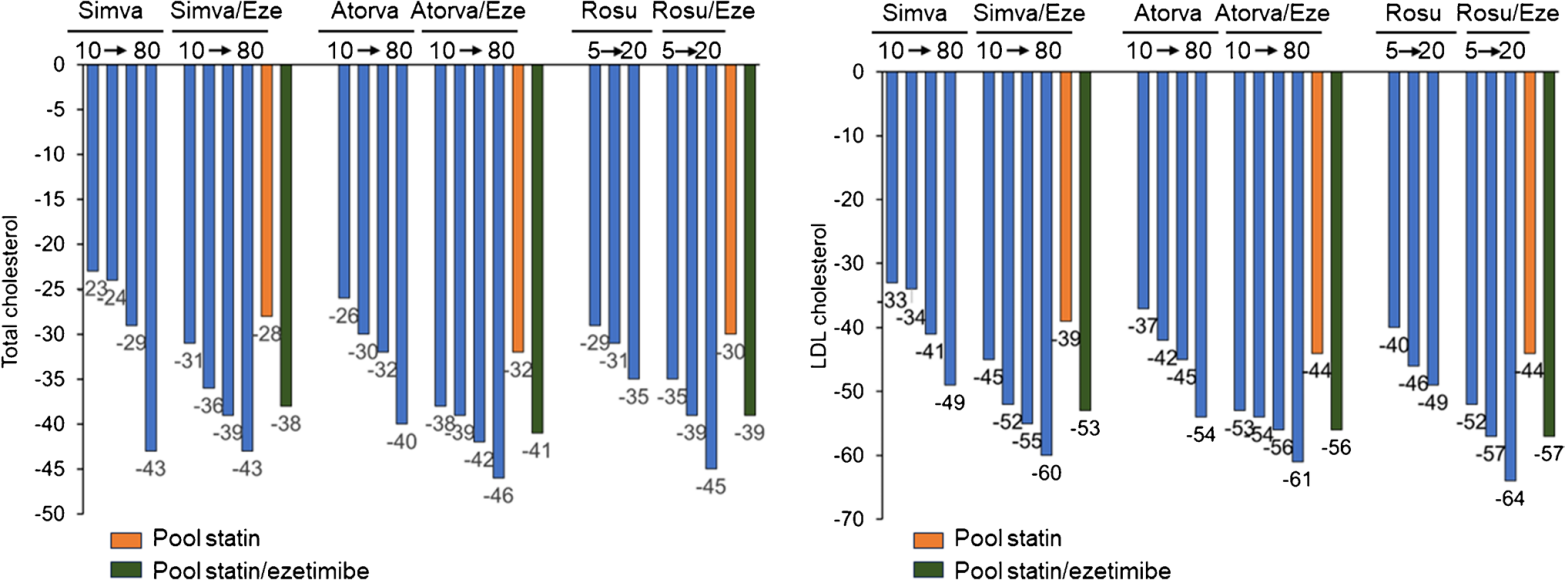
Lipid-lowering effect of ezetimibe (10 mg) in combination with statins. (Modified with permission from: De Luca L. et al. Kardiologia polska. 2020;78:850-860) [28]

The addition of ezetimibe to statins can provide an adequate strategy to reach the LDL-C goal avoiding the use of high-intensity statin monotherapy with potential higher incidence of muscle-related side effects (Fig. 1) [28].

In the IMPROVE-IT study, the efficacy and safety of the combination simvastatin/ezetimibe (40 mg/10 mg) was compared with simvastatin (40 mg) in patients who had been hospitalized for an acute coronary syndrome (ACS) and had LDL-C levels of 50 to 100 mg/dL (under lipid-lowering therapy) or 50 to 125 mg/dL (without lipid-lowering therapy) [29•]. This was the first trial demonstrating that ezetimibe, when added to statin therapy, further reduced the LDL-C levels (−24% compared to simvastatin monotherapy) and reduced by 2% the absolute primary end point at 7 years (composite of cardiovascular death, nonfatal myocardial infarction, unstable angina requiring rehospitalization, coronary revascularization improved cardiovascular outcomes) [29•]. The effect was mainly driven by reduction in myocardial infarction (hazard ratio 0.87) and ischemic stroke (hazard ratio 0.79). These results occurred irrespectively of baseline LDL-C values, were proportional to the difference in LDL-C concentrations between the groups and supported the use of intensive lipid-lowering therapy with ezetimibe even in patients with baseline LDL-C <70 mg/dL [30]. These effects occurred without indications that ezetimibe increased risk of adverse events including new-onset diabetes mellitus [31].

Recently, a randomized, open-label, non-inferiority trial conducted in South Korea showed that in patients with ASCVD, the combination of moderate-intensity statin with ezetimibe (10 mg rosuvastatin) was non-inferior to high-intensity statin monotherapy (20 mg rosuvastatin) in terms of cardiovascular outcomes (a composite of cardiovascular death, major cardiovascular events, or non-fatal stroke) [32]. In addition, the non-inferiority of 3-year clinical outcomes was achieved with a higher proportion of LDL-C levels below 70 mg/dL and a lower incidence of discontinuation or dose reduction caused by intolerance to the study drugs [32]. These conclusions were confirmed when the analysis was restricted to those who underwent percutaneous coronary intervention (PCI) [33].

In the PRECISE-IVUS study conducted in patients who underwent PCI, the addition of ezetimibe to atorvastatin showed a greater reduction of LDL-C than atorvastatin monotherapy and a significantly higher coronary plaque regression with negative vascular remodelling in the analysed target segment [34]. These results confirm that the combination therapy with statin plus ezetimibe is a valuable lipid-lowering option for high-risk patients.

The development of fixed drug combinations requires the evaluation of the bioequivalence with co-administration of the separate reference products, i.e. ezetimibe and rosuvastatin. A single-dose, randomized, two-way crossover bioequivalence study has been conducted for comparing the test product (rosuvastatin/ezetimibe, 40 mg/10 mg) with the reference ones (rosuvastatin 40 mg, and ezetimibe 10 mg). The AUC0-∞ and C_max_ for both rosuvastatin and ezetimibe in combination were within the confidence intervals demonstrating their bioequivalence, which indicated that their efficacy and safety profile can be considered the same as for the monocomponents [35].

A greater improvement in lipid-lowering effect with the statins/ezetimibe combination was observed by comparing the effectiveness of fixed-dose combinations vs separate pills [36••]. A retrospective analysis, using electronic medical records of outpatients at very-high CV risk, showed that the addition of ezetimibe in patients already under statin treatment further reduced LDL-C by 19.4% while fixed-dose combination determined a greater reduction of 28.4% [36••]. Although this study did not provide the underlying cause of improved LDL-C lowering with fixed drug combination, a better medication adherence is a likely explanation. Indeed, the analysis of a large Italian cohort of patients newly treated with statins to whom ezetimibe was additionally administered, observed that those prescribed a single-pill combination had 87% greater odds of being highly adherent compared to those treated with a two-pill combination [37]. Considering this, the European guidelines recommend the combination with ezetimibe if the LDL-C goals are not achieved with the maximum tolerated statin dose (class of recommendation I and level of evidence B) [2]. A simulation model with a five-year horizon (2020–2024) across six countries estimated that treatment intensification with strategies based on statin, ezetimibe, and statin plus ezetimibe in fixed drug combination results in substantial benefit in terms of LDL-C goal achievement and major adverse coronary events (MACE) reduction compared to status quo treatment [38]. Intervention with statins and ezetimibe as separate pills or in combination resulted in estimated relative MACE reduction by 5.4 and 6.4% representing ∼3.7 and 4.4 million MACE averted, respectively [38].

## Fixed Combination of Bempedoic Acid and Ezetimibe

Bempedoic acid is a prodrug which undergoes to liver-specific activation by very long-chain acyl-CoA synthetase-1 (ACSVL1) to bempedoic acid-CoA which is a competitive inhibitor of the ACLY [39]. Since ACSVL1 enzyme is selectively expressed in the liver, bempedoic acid is inactive in extrahepatic organs including the skeletal muscles, and thus may not determine muscle-related adverse effects [40••]. Similarly, to statins, bempedoic acid reduces the endogenous synthesis of cholesterol, thus, for this reason, its combination with ezetimibe represents an effective intervention for controlling dyslipidaemia. Bempedoic acid (180 mg) is currently available as monotherapy or as fixed oral combination with ezetimibe (10 mg). Bempedoic acid with a single daily dose reduces LDL-C by a mean 24.5% when given alone, by 18% when given on top of a major statin and by 38–40% when given in a fixed-dose combination with ezetimibe [41].

After oral administration, bempedoic acid is absorbed from the small intestine with a T_max_ of 3.5 h, and its major route of elimination is via metabolism to acyl glucuronide [42]. At steady state, the mean half-life of bempedoic acid in humans is 19 h. The pharmacokinetic of bempedoic acid is minimally altered in people with mild to moderate renal impairment [42]. Bempedoic acid and its glucuronides are weak inhibitors of the transporter proteins organic anion-transporting polypeptide 1B1 (OATP1B1) and 1B3 (OATP1B3) involved in the liver uptake of statins. Indeed, this drug is contraindicated with simvastatin at doses higher than 40 mg.

After completion of four phase 3 trials, data on the cardiovascular outcome trial have been recently published [43••]. In the CLEAR-Outcomes study after a follow-up of 40.6 months, in statin-intolerant patients, bempedoic acid was superior to placebo to reduce the incidence of MACE (hazard ratio, 0.87; 95%CI 0.79–0.96), as were the incidences of a composite of death from cardiovascular causes, nonfatal stroke, or nonfatal myocardial infarction (hazard ratio, 0.85; 95%CI, 0.76–0.96); fatal or nonfatal myocardial infarction (hazard ratio, 0.77; 95%CI, 0.66–0.91); and coronary revascularization (hazard ratio, 0.81; 95%CI, 0.72–0.92). Among the 6992 patients treated with bempedoic acid, 11.5% were also on ezetimibe [43••]. The cardiovascular benefit of bempedoic acid was even more pronounced in the subgroup of high-risk primary prevention patients [44]. Of importance similarly to statins, ezetimibe and PCSK9 inhibitors [45] benefits were proportional to the difference in LDL-C achieved between study groups [43••].

Phase 2 clinical study in patients with type 2 diabetes and hypercholesterolaemia not treated with statins, demonstrated that, after 12 weeks of treatment, the fixed combination bempedoic acid/ezetimibe lowered mean LDL-C by 38.8%, which was significantly greater than with ezetimibe alone (19.2%). Significantly more patients achieved LDL-C levels < 70 mg/dL when treated with fixed combination bempedoic acid/ezetimibe (38.9%) than did patients taking ezetimibe (5.4%) [46••]. Of patients who were treated with fixed bempedoic acid/ezetimibe combination, 40.7% achieved a reduction in LDL-C of ≥ 50% from baseline [46••]. At week 12, fixed combination therapy reduced median hsCRP by 25.3%, which was significantly greater than with ezetimibe alone (increased by 2.1%). Differently from previous evidence with statins, bempedoic acid did not increase glycaemia and new-onset diabetes [46••] and was not associated to muscle-related side effects [47]. Similar data were observed in the phase 3 clinical trial CLEAR Tranquility where statin intolerant patients were treated with bempedoic acid added to background lipid-modifying therapy that included ezetimibe. The addition of bempedoic acid reduced LDL-C by 28.5% more than placebo together with −23.6% reductions of non-HDL-C, −18% of TC, −19.3% of Apo-B, and −31.0% of hs-CRP [48].

The LDL-C–lowering effect of the fixed-dose combination bempedoic acid/ezetimibe was confirmed also in statin-treated patients with a −36.2% reduction vs placebo compared to −23.2% and −17.2% of ezetimibe or of bempedoic acid alone respectively [49]. Interestingly, the fixed-dose combination lowered LDL-C levels similarly across patients receiving high-intensity, other-intensity or no statin therapy [49].

Taken together, prescription of bempedoic acid may be useful upon the observation of statin-associated muscular side effects, which may be found in roughly 5% of statin-treated patients [50]. In this case, an agent associated with a very low risk of these symptoms appears attractive.

## Fixed Combination of Statins and Fenofibrate

In the fasting state, circulating triglycerides (TG) are mainly transported by the very low-density lipoprotein (VLDL), and with their remnants, these particles represent part of circulating ApoB-containing lipoproteins. Elevated plasma TG levels are associated with an increasing risk of ASCVD, but this association becomes null after adjusting for non-HDL-C [51]. Non-HDL-C includes all ApoB-containing lipoproteins and is calculated by subtracting the HDL-C from TC concentrations. The association between non-HDL-C and CV risk is at least as strong as the one with LDL-C [52].

A Mendelian randomization study demonstrated that lipoprotein lipase (LPL) genetic variants associated to low TG levels have the same association with ASCVD risk as LDL receptor variants determining a reduction in plasma LDL-C. Both groups of variants had the same effect on the risk of ASCVD per unit change of ApoB, indicating that all ApoB-containing lipoproteins have the same effect on the risk [53]. Together, these data indicate that the concentration of ApoB-containing particles, rather than their TG content itself, has a causal effect on ASCVD. Thus, a therapy that may reduce the TG levels, but most importantly the number of ApoB-containing lipoproteins, may protect against CVD events. In addition, the combination of TG-lowering therapies with hypocholesterolemic drugs may show a protective additive effect especially in patients with diabetes with mixed dyslipidaemia characterized by a triad of hypertriglyceridemia, low plasma concentrations of HDL, and qualitative changes in LDL.

Fibric acid derivatives represent the most used therapeutic option for controlling TG levels and include many chemical entities (clofibrate, gemfibrozil, fenofibrate, bezafibrate, ciprofibrate and pemafibrate) although fenofibrate is by far the most utilized in clinical practice. Fibrates reduce TG levels by activating the peroxisome proliferator-activated receptor α (PPARα) and stimulating fatty acid oxidation, increasing LPL synthesis, and reducing the expression of ApoC III. By increasing LPL synthesis and reducing the LPL inhibitor ApoC III, fibrates enhance the clearance of TG-rich lipoproteins. The increase in HDL is, instead, due to PPARα stimulation of apo A-I and apo A-II expression, and via a decrease of cholesteryl ester transfer protein (CETP) activity that transfers cholesterol from HDL to VLDL [54, 55].

Fenofibrate is a pro-drug transformed into its active form, fenofibric acid, in the liver. Fenofibrate has a bioavailability of 60%, a T_max_ of 3.5 h, and a half-life time of 20 h. Fenofibrate is excreted predominantly as glucuronide conjugates (60%) in the urine, with smaller amounts appearing in the feces [54]. While gemfibrozil inhibits hepatic uptake of statins by OATP1B1 and competes for the same glucuronosyl transferases that metabolize most statins determining a clinically relevant drug interaction, fenofibrate is glucuronidated by enzymes that are not involved in statin glucuronidation. Thus, fenofibrate-statin combinations are less likely to cause myopathy than combination therapy with gemfibrozil and statins.

The clinical effects of fibrates have been tested in six randomized control trials including the ACCORD (Action to Control Cardiovascular Risk in Diabetes) trial where fenofibrate was added to statin therapy [56]. Overall, the reduction in CVD outcomes by fibrates appeared to be proportional to the degree of non-HDL-C lowering [24], although neither the FIELD nor the ACCORD studies involving fenofibrate reached their primary outcome [56, 57]. Results from meta-analyses suggest reduced major CVD events, e.g., coronary heart disease (10–13%) in patients with high TGs and low HDL-C in fibrate-treated patients, but no decrease in strokes, CVD or total mortality [58–60]. Thus, the overall efficacy of fibrates on CVD outcomes is much less robust than that of statins.

Recently, a new selective PPAR-α modulator pemafibrate has been reported to have marked efficacy in reducing TGRL [61]. The PROMINENT study was specifically designed to test the hypothesis that PPARα agonists have a clinical benefit mainly in patients with high TG (between 200 and 499 mg/dL) and low HDL-C levels (40 mg/dL or less) and particularly with concomitant type 2 diabetes and in use of statins [62•]. At baseline, 95.7% of patients were receiving statin therapy and baseline median TG, LDL-C and HDL-C were respectively 271 mg/dL, 78 mg/dL and 33 mg/dL. Pemafibrate reduced TG levels by 26.2%, these between-group differences were −24.6% in patients who received a high-intensity statin, −28.5% in those who received a moderate-intensity statin, and −34.3% in those who had minimal statin use. Nevertheless, the major adverse cardiovascular events (a composite of myocardial infarction, ischemic stroke, hospitalization for unstable angina warranting unplanned coronary revascularization, or death from cardiovascular causes) was similar between the pemafibrate and placebo group. Thus, pemafibrate did not reduce the incidence of cardiovascular events despite a significant reduction (26–28%) of TG, VLDL cholesterol (−26%), remnant cholesterol (−25.6%), and ApoC III (−27.6%) levels [62•]. Of importance, pemafibrate therapy increased LDL-C and Apo-B by 12.3% and 4.3%. The increment in LDL-C and absent reduction in Apo-B containing lipoproteins clearly indicate that even if there is favourable remodelling in TG-rich particles but without reduction in particle number per se, represented by ApoB concentrations there will be no benefit from TG lowering therapies [63, 64]. These neutral findings are consistent with those from the FIELD [65] and ACCORD [56] trials with fenofibrate, all of which enrolled patients with high TG levels. Except for the FIELD trial, all the other trials involved patients who were receiving statins.

The fixed-combination fenofibrate/atorvastatin (100 mg/40 mg) demonstrated a significantly greater reduction in TG and non-HDL-C compared with atorvastatin and fenofibrate alone, respectively. These findings, after 12 weeks of treatment, suggest that the fixed-combination provided effective management of lipids that was consistent with the actions of the individual drugs [66]. This treatment was generally well tolerated and supported the use of the combination for a better control of lipid profile with a potential improvement of adherence. Very similar results were observed with the fixed combination simvastatin/fenofibrate (20/160 mg) in the DIACOR (Diabetes and Combined Lipid Therapy Regimen) study that enrolled diabetic patients [67], and in the SAFARI (Effectiveness and tolerability of simvastatin plus fenofibrate for combined hyperlipidaemia) study [68]. The fixed combinations were shown an improved efficiency of lipid lowering relative to coadministration studies that enrolled similar patients with mixed hyperlipidaemias, without any cases of myositis, myalgia, or rhabdomyolysis, which as observed in coadministration study [69]. Significant differences between combination fenofibrate/simvastatin and simvastatin monotherapies were also observed in patient at high and very high CVD risk for the percentage change in TG levels (mean difference −32.2%), HDL-C (mean difference +7.5%), and LDL-C (mean difference −34.7%) [70]. Thus, fenofibrate/simvastatin combinations are considered effective and well-tolerated therapies to improve the TG and LDL-C profile in high CV risk patients.

In 2016, the Food and Drug Administration (FDA) withdrew approval for use of fenofibrate in addition to statin therapy for ASCVD risk reduction (https://www.federalregister.gov/articles/2016/04/18/2016-08887/abbvie-inc-et-al-withdrawal-of-approval-of-indications-related-to-the-coadministration-with-statins). However, the current European Society of Cardiology (ESC) and European Atherosclerosis Society (EAS) guidelines that were issued before PROMINENT study results suggest, with class of recommendation IIb and level of evidence C, that fenofibrate or bezafibrate may be considered in combination with statins on high-risk patients who are at LDL-C goal with TG levels >2.3 mmol/L (>200 mg/dL) [2]. These recommendations will probably be reviewed to class III for ASCVD prevention after the negative results of PROMINENT [63].

## Conclusions

**Fig. 2:**
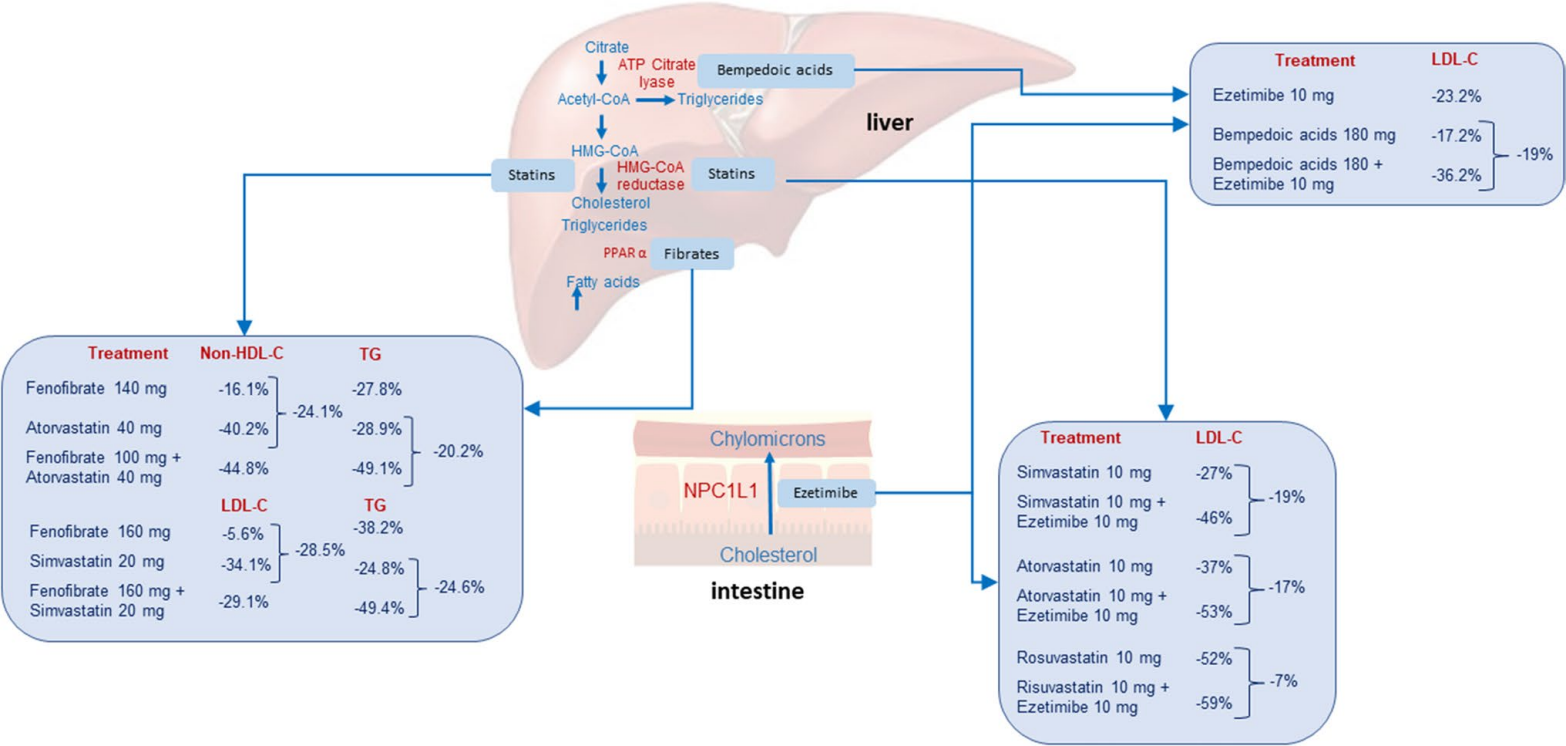
Schematic representation of fixed combination therapies, their mechanism of action and lipid-lowering effects. Values were from Ballantyne CM et al., Davidson MH et al., Muhlestein JB et al., Ballantyne CM et al., and Kim KJ [49, 66, 67, 71, 72]

Fixed-dose combinations are currently becoming the standard and, very often, the first line of treatment for an efficient and safe pharmacological control of CVD risks, including the one ascribed to LDL-C. By using two drugs acting with different or the same metabolic pathways, it is possible to achieve LDL-C goals by avoiding the maximum monotherapy doses of either drugs, thus decreasing the prevalence of dose-dependent adverse events. Single-pill combination therapies may also improve medication adherence. Currently, the combinations statins/ezetimibe and bempedoic acid/ezetimibe represent the established and the new frontier in controlling LDL-C levels by inhibiting cholesterol biosynthesis and gastrointestinal absorption. Their hypolipidemic effect is somewhat additive (Fig. 2). On the contrary, fenofibrate and statins are acting on distinct metabolic pathways controlling cholesterol and TG levels, respectively (Fig. 2). Their combination was shown to produce an effective hypolipidemic action in diabetic and high CVD risk patients with mixed dyslipidaemia. However, the negative results of the PROMINENT [62•] study cast severe doubts on the utility of this combination for reduction of ASCVD events [63]. Finally, a broader approach for controlling CVD may consider the combination of therapies acting on different modifiable factors, such as LDL-C, blood pressure and thrombosis. The polypill may be useful in the prevention of CVD by improving cardiovascular risk factors and increasing compliance with drug therapy. Treatment with fixed combination containing aspirin, ramipril, and atorvastatin, within 6 months after myocardial infarction was shown to significantly lower risk of major adverse cardiovascular events compared to usual care [16•].
